# Supplementary material for: Clinically relevant gene signatures provide independent prognostic information in older breast cancer patients
Source: Breast Cancer Res. 2024 Mar 7;26:38. doi: 10.1186/s13058-024-01797-7 (PMC10921680; doi:10.1186/s13058-024-01797-7)
Supplement: Supplementary file 2 — Additional file 2. Supplementary Figures 1-3. [file 13058_2024_1797_MOESM2_ESM.pdf]

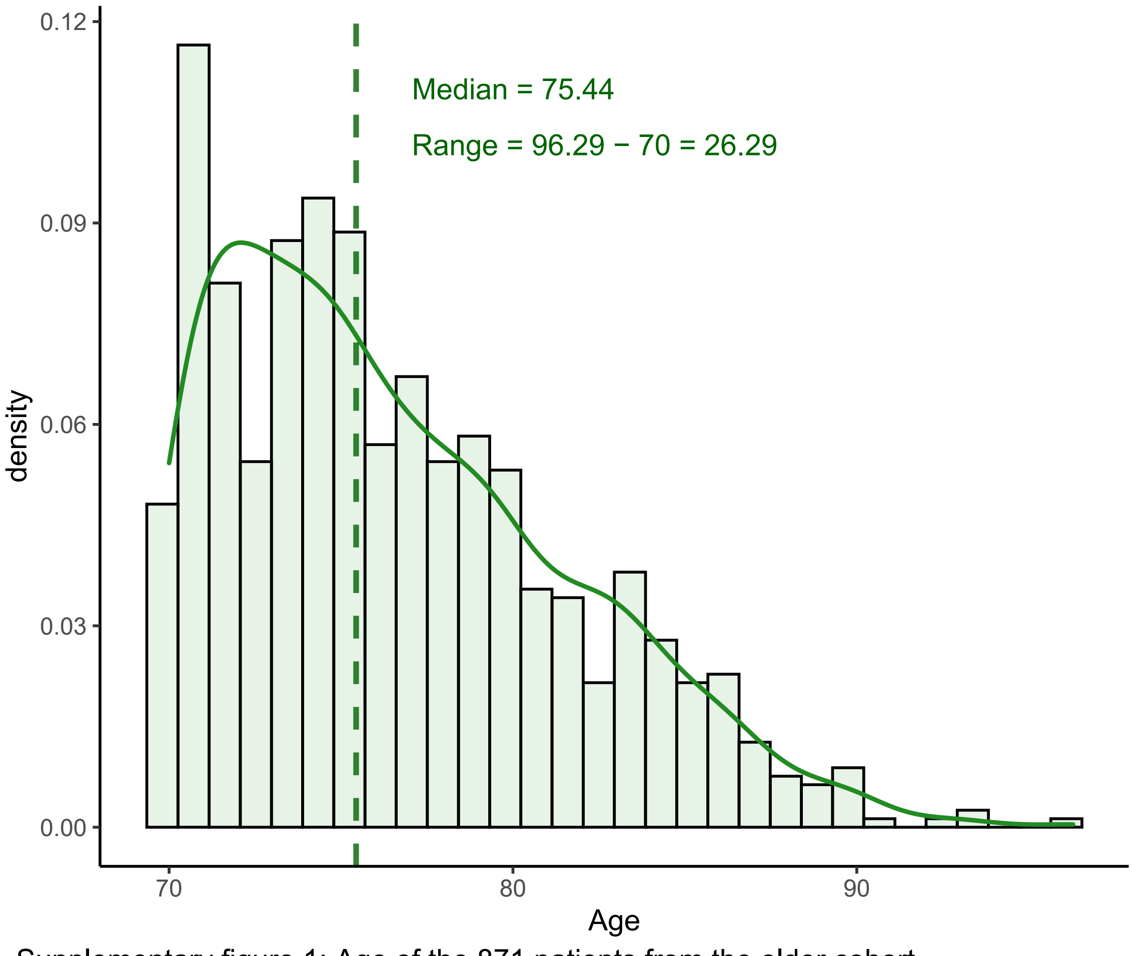

Supplementary figure 1: Age of the 871 patients from the older cohort.

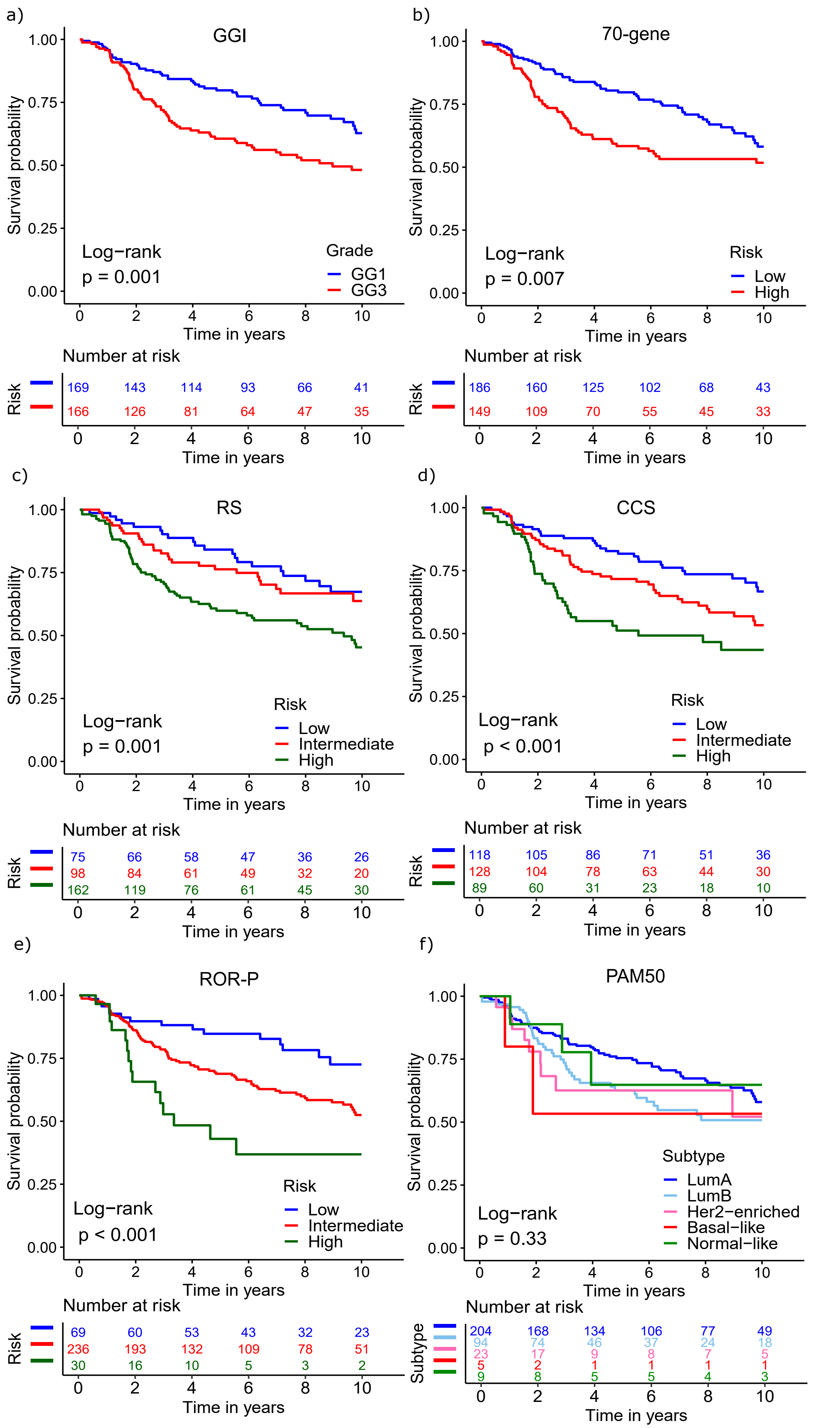

Supplementary Figure 2: Kaplan-Meier analysis of gene expression signatures in the ER+/LN+ patients. (a) Genomic Grade Index (GGI) (b) 70-gene (c) Recurrence score (RS) (d) Cell-cycle score (CCS) (e) PAM50 Risk of Recurrence score - Proliferation (ROR-P) (f) Prediction Analysis of Microarray 50 (PAM50).

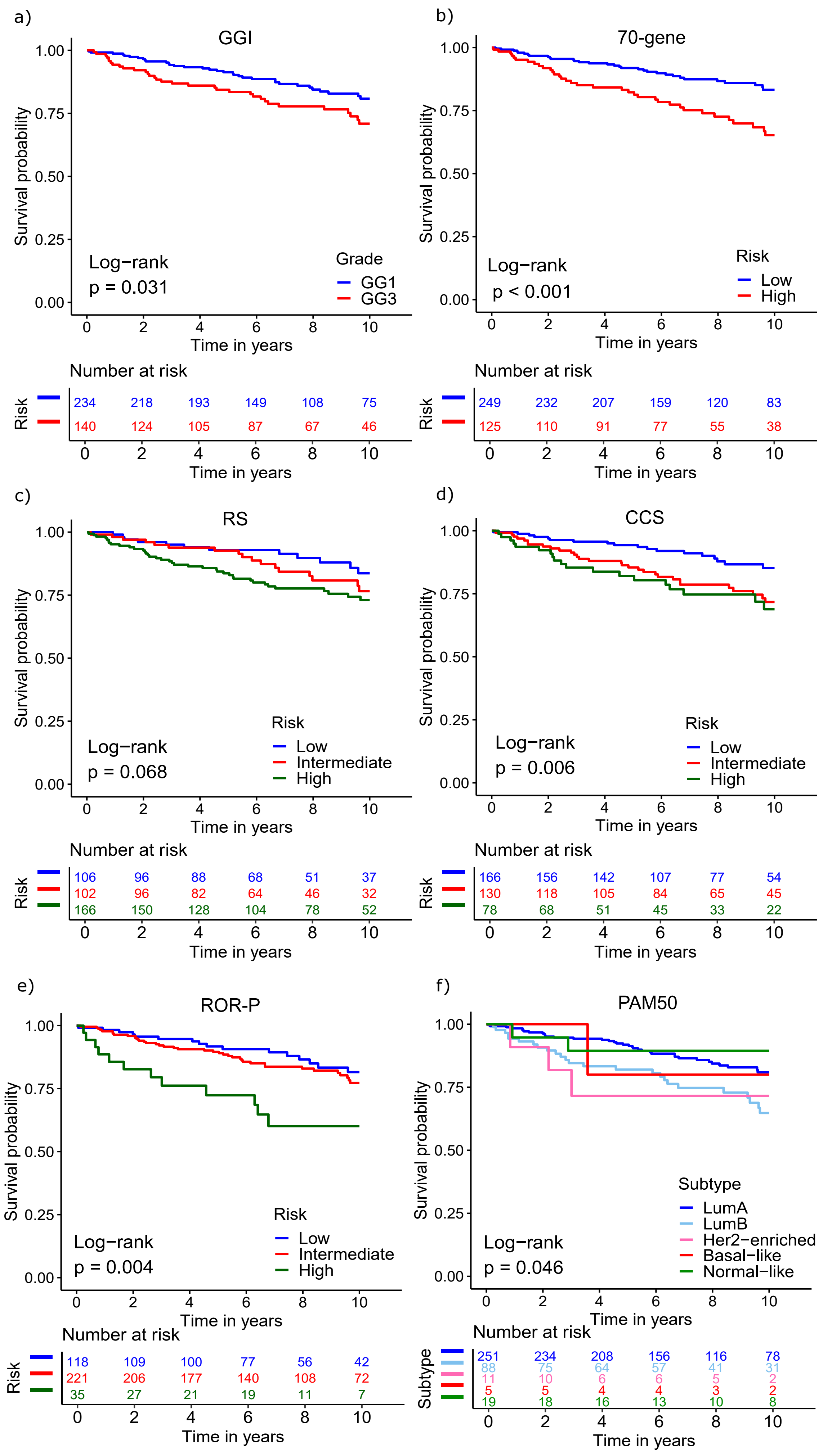

Supplementary Figure 3: Kaplan-Meier analysis of gene expression signatures in the ER+/LN- patients. (a) Genomic Grade Index (GGI) (b) 70-gene (c) Recurrence score (RS) (d) Cell-cycle score (CCS) (e) PAM50 Risk of Recurrence score - Proliferation (ROR-P) (f) Prediction Analysis of Microarray 50 (PAM50).
