# Supplementary material for: Clinically relevant gene signatures provide independent prognostic information in older breast cancer patients
Source: Breast Cancer Res. 2024 Mar 7;26:38. doi: 10.1186/s13058-024-01797-7 (PMC10921680; doi:10.1186/s13058-024-01797-7)
Supplement: Supplementary file 3 — Additional file 3. Supplementary Tables 2-7. [file 13058_2024_1797_MOESM3_ESM.pdf]

Supplementary table 2: Gene signature risk stratification of elderly patients.

	Patients ≥ 70 (N = 871)
Signature	*N(%)*
GGI	
GG1	453 (52)
GG3	418 (48)
70-gene	
Low risk	479 (55)
High risk	392 (45)
Recurrence score	
Low risk	199 (23)
Intermediate risk	211 (24)
High risk	461 (53)
Cell cycle score	
Low	329 (38)
Intermediate	283 (32)
High	259 (30)
RORP	
Low proliferation	206 (24)
Intermediate proliferation	532 (61)
High proliferation	133 (15)
PAM50	
Luminal A	493 (57)
Luminal B	206 (24)
Her2-enriched	69 (8)
Basal-like	65 (7)
Normal-like	38 (4)

Supplementary Table 3: Multivariable analysis for all the elderly patients, ER+/LN-/HER2- patients for GGI, 70-gene, RS, CCS, ROR-P, and PAM50 signatures. We used RFS as the clinical endpoint.

		Patients (ER+/LN-/HER2-)* (N = 222)	
Signature	*N(%)*	*HR (95% CI)*	*P*
Genomic grade index			
GG1 (ref)	131 (59)	1 (-)	-
GG3	91 (41)	1.6 (0.8 - 3.3)	0.17
70-gene			
Low risk (ref)	149 (67)	1 (-)	-
High risk	73 (33)	2.3 (1.2 - 4.4)	**0.02**
Recurrence score			
Low risk (ref)	59 (26)	1 (-)	-
Intermediate risk	77 (35)	0.9 (0.3 - 2.2)	0.75
High risk	86 (39)	1.3 (0.6 - 3.2)	0.5
Cell cycle score			
Low (ref)	102 (46)	1 (-)	
Intermediate	76 (34)	2.2 (1 - 4.8)	0.05
High	44 (20)	2.4 (1 - 5.9)	0.06
RORP			
Low proliferation (ref)	65 (30)	1 (-)	-
Int. proliferation	142 (64)	1.4 (0.6 - 3.2)	0.38
High proliferation	15 (6)	1.5 (0.3 - 6.2)	0.6
PAM50			
Luminal A (ref)	152 (69)	1 (-)	-
Luminal B	53 (24)	1.7 (0.8 - 3.5)	0.15
Her2-enriched	5 (2)	8.9 (2.2 - 35.3)	**0.002**
Basal-like	2 (1)	0 (0 - Inf)	1
Normal-like	10 (4)	0 (0 - Inf)	1

NOTE: Bold values indicate P < 0.05.

* Adjusted for tumor size, tumor grade, and hormonal therapy.

Supplementary table 4: Additional prognostic value of gene signatures based on delta likelihood ratio (ΔLR) on top of tumor grade, tumor size, and estrogen and lymph node status for all patients, and on top of tumor grade and tumor size for ER+/LN+ and ER+/LN-.

	All patients[a] (N = 871)			Patients (ER+/LN+)[b] (N = 335)			Patients (ER+/LN-)[b] (N = 374)		
Signature	ΔLR	*P*	*c-index*	ΔLR	*P*	*c-index*	ΔLR	*P*	*c-index*
Genomic grade index	6.24	**0.01**	0.67	6.67	**0.01**	0.62	1.43	0.23	0.64
70 gene	11.47	**<0.001**	0.68	5.84	**0.01**	0.64	6.18	**0.01**	0.66
Recurrence score	14.11	**<0.001**	0.68	13.59	**<0.01**	0.64	1.84	0.39	0.65
Cell cycle score	16.97	**<0.001**	0.69	13.19	**<0.01**	0.65	6.49	**0.04**	0.67
RORP	15.38	**<0.001**	0.68	15.82	**<0.001**	0.64	5.25	0.07	0.65
PAM50	9.57	**0.05**	0.67	4.14	0.38	0.61	7.89	0.09	0.65

NOTE: Bold values indicate P < 0.05.

[a] Adjusted for tumor size, tumor grade, estrogen receptor status, lymph node status, and hormonal therapy.

[b] Adjusted for tumor size, tumor grade, and hormonal therapy.

Supplementary table 5: Clinico-pathological characteristics of the ER+/LN- patients between 55 and 65 years old.

	Patients (N = 478)	
	Number	Percent
Primary tumor characteristics		
Progesterone receptor status		
Positive	219	45.8
Negative	113	23.6
Unknown	146	30.6
HER2		
Positive	30	6.3
Negative	272	56.9
Unknown	176	36.8
Elston-Ellis tumor grade		
1	78	16.3
2	201	42.0
3	160	33.5
Unknown	39	8.2
Tumor size (cm)		
<2	228	47.7
≥2	219	45.8
Unknown	31	6.5
Treatment		
Chemotherapy	1	0.2
Hormonotherapy	156	32.6
Chemotherapy + Hormonotherapy	18	3.8
Untreated	257	53.8
Unknown	46	9.6

Supplementary Table 6: Multivariable analysis for ER+/LN- patients between 55 and 65 years for GGI, 70-gene, RS, CCS, ROR-P, and PAM50 signatures. We used RFS as the clinical endpoint.

‘

		Patients 55-65 (ER+/LN-)* (N = 478)			
Signature	*N(%)*	*HR (95% CI)*	*P*	*ΔLR*	*P*
Genomic grade index					
GG1 (ref)	268 (56)	1 (-)	-	6.23	**0.01**
GG3	210 (44)	1.8 (1.1 - 2.8)	**0.01**		
70-gene					
Low risk (ref)	297 (62)	1 (-)	-	10.11	**<0.01**
High risk	181 (38)	2 (1.3 - 3.1)	**<0.01**		
Recurrence score					
Low risk (ref)	118 (25)	1 (-)	-		
Intermediate risk	115 (24)	1.4 (0.7 - 2.8)	0.39	6.59	**0.04**
High risk	245 (51)	2.1 (1.1 - 3.9)	**0.02**		
Cell cycle score					
Low (ref)	200 (42)	1 (-)			
Intermediate	158 (33)	2.1 (1.2 - 3.7)	**<0.01**	8.93	**0.01**
High	120 (25)	2.1 (1.2 - 3.9)	**0.01**		
RORP					
Low proliferation (ref)	162 (34)	1 (-)	-		
Intermediate proliferation	246 (51)	2.6 (1.5 - 4.8)	**<0.01**	12.02	**<0.01**
High proliferation	70 (15)	2.1 (1 - 4.5)	0.053		
PAM50					
Luminal A (ref)	283 (59)	1 (-)	-		
Luminal B	132 (28)	1.6 (1 - 2.6)	0.051		
Her2-enriched	27 (5)	1.3 (0.6 - 2.7)	0.56	4.58	0.33
Basal-like	14 (3)	1.9 (0.7 - 5.4)	0.22		
Normal-like	22 (5)	1 (0.3 - 3.2)	0.97		

NOTE: Bold values indicate P < 0.05.

* Adjusted for tumor size, tumor grade, and hormonal therapy.

Supplementary table 7: Gene signature risk stratification of ER+/LN- elderly patients and patients between 55 and 65 years old.

Signature	Patients ≥ 70 (N = 374) *N(%)*	Patients (55 ≤ age ≤ 65) (N = 478) *N(%)*	*P*
GGI			
GG1	234 (63)	268 (56)	0.06
GG3	140 (37)	210 (44)	
70-gene			
Low risk	249 (67)	297 (62)	0.20
High risk	125 (33)	181 (38)	
Recurrence score			
Low risk	106 (29)	118 (25)	
Intermediate risk	102 (27)	115 (24)	0.14
High risk	166 (44)	245 (51)	
Cell cycle score			
Low	166 (44)	200 (42)	
Intermediate	130 (35)	158 (33)	0.34
High	78 (21)	120 (25)	
RORP			
Low proliferation	118 (31)	162 (34)	
Intermediate proliferation	221 (59)	246 (51)	**0.02**
High proliferation	35 (10)	70 (15)	
PAM50			
Luminal A	251 (67)	283 (59)	
Luminal B	88 (24)	132 (28)	
Her2-enriched	11 (3)	27 (5)	0.052
Basal-like	5 (1)	14 (3)	
Normal-like	19 (5)	22 (5)	

NOTE: Correlations were calculated using χ^2 test unless otherwise specified. Bold values indicate P < 0.05.
